# Adverse-Pressure-Gradient Effects on Turbulent Boundary Layers: Statistics and Flow-Field Organization

**DOI:** 10.1007/s10494-017-9869-z

**Published:** 2017-11-10

**Authors:** Carlos Sanmiguel Vila, Ramis Örlü, Ricardo Vinuesa, Philipp Schlatter, Andrea Ianiro, Stefano Discetti

**Affiliations:** 1 Aerospace Engineering Group, Universidad Carlos III de Madrid, Leganés, Spain; 2 Linné FLOW Centre, KTH Mechanics, SE-100 44 Stockholm, Sweden

**Keywords:** Wall turbulence, PTV, PIV, POD

## Abstract

This manuscripts presents a study on adverse-pressure-gradient turbulent boundary layers under different Reynolds-number and pressure-gradient conditions. In this work we performed Particle Image Velocimetry (PIV) measurements supplemented with Large-Eddy Simulations in order to have a dataset covering a range of displacement-thickness-based Reynolds-number 2300 $<Re_{\delta ^{*}}<$ 34000 and values of the Clauser pressure-gradient parameter *β* up to 2.4. The spatial resolution limits of PIV for the estimation of turbulence statistics have been overcome via ensemble-based approaches. A comparison between ensemble-correlation and ensemble Particle Tracking Velocimetry was carried out to assess the uncertainty of the two methods. The effects of *β*, *R* *e* and of the pressure-gradient history on turbulence statistics were assessed. A modal analysis via Proper Orthogonal Decomposition was carried out on the flow fields and showed that about 20% of the energy contribution corresponds to the first mode, while 40% of the turbulent kinetic energy corresponds to the first four modes with no appreciable dependence on *β* and *R* *e* within the investigated range. The topology of the spatial modes shows a dependence on the Reynolds number and on the pressure-gradient strength, in line with the results obtained from the analysis of the turbulence statistics. The contribution of the modes to the Reynolds stresses and the turbulence production was assessed using a truncated low-order reconstruction with progressively larger number of modes. It is shown that the outer peaks in the Reynolds-stress profiles are mostly due to large-scale structures in the outer part of the boundary layer.

## Introduction

The quest for a better understanding of turbulent boundary layers (TBLs) is one of the main research goals of the turbulence community since many decades, as stated for instance in Ref. [1]. Wall-bounded turbulence is present in many relevant fluid-flow problems such as the flow around wings, land and sea vehicles, or in turbines, compressors, etc. Simplified scenarios, such as the zero-pressure-gradient (ZPG) TBL developing over a flat plate, have been investigated to understand the fundamental aspects of wall-bounded turbulence. Unfortunately, ZPG conditions are nearly never encountered in real-life applications; instead, the majority of flow problems are under the effect of complex pressure gradients. In particular, adverse pressure gradients might produce flow separation with the consequent losses in performances. Under these conditions, the applicability of the knowledge from ZPG TBLs to decelerating boundary layers is still rather limited [2, 3]. Despite the existence of a number of simulations and experiments on adverse-pressure-gradient (APG) TBLs (among many others, see e.g. Refs. [2–9]), there is still no clear understanding of the isolated effects of the imposed pressure-gradient, of its upstream history and of the Reynolds number. The wider parametric space with respect to ZPG TBLs and the importance of history effects in the development of the flow are some of the reasons which make the study of these flows challenging. In an attempt to reduce the number of parameters which characterize the history effects, most of the APG studies are performed in a state of near-equilibrium. This implies that the mean velocity deficit in the outer part is self-similar at sufficiently high Reynolds numbers as discussed, among others, in Ref. [1]. The streamwise evolution of the free-stream velocity *U*_*∞*_(*x*) in an APG TBL under near-equilibrium conditions follows a power-law relation such that *U*_*∞*_ = *C*(*x* − *x*_0_)^*m*^. Here *C* is a constant, *x*_0_is a virtual origin and the exponent *m* ranges between − 1/3 < *m* < 0[10].

Some important features of APG flows have already been clarified in the past decades. The most recognizable feature of an APG TBL is the more prominent wake of streamwise mean velocity profile [11]. The strengthened wake reflects the local state of the boundary layer as a consequence of the impact of history effects experienced by the flow. The wake strength is connected to the Clauser pressure-gradient parameter *β* [12], which is defined as *β* = (*δ* ^∗^/*τ*_*w*_)(d*P*/d*x*), where *δ* ^∗^ is the displacement thickness, *τ*_*w*_ is the mean wall-shear stress, and d*P*/d*x* is the derivative of the static pressure along the streamwise coordinate. As *β* increases, the mean velocity profile develops a larger wake region and the streamwise variance profile exhibits an outer peak, which is related to the development of more energetic large-scale motions [3]. The appearance of more energetic structures in the outer region is also accompanied by larger values of the inner peak of the streamwise variance profile [13, 14].

On the other hand, there is some controversy on whether the logarithmic law of the wall still holds in APG TBL flows [15, 16]. There are studies where it is claimed that the law of the wall is still valid, but that the region occupied by the logarithmic law is progressively reduced when the pressure gradient is increased. Furthermore some studies report that the logarithmic region shifts with increasing pressure gradient strength below the one for canonical ZPG TBLs [11, 17]. The streamwise velocity profile normalized with respect to the friction velocity is below the ZPG profile in the buffer region for progressively stronger APGs. Consequently the *U* ^+^ slope is found to increase with increasing APG, leading to lower values of the von Kármán constant *κ*[2, 18]. Some authors, on the other hand, propose a dependence of the constants in terms of the pressure-gradient parameter in inner units $p_{x}^{+} =(\nu /\rho u_{\tau }^{3})(dP/dx)$ [19]. In other works it is argued that the existence of the law of the wall is conditioned to the near-equilibrium state [20].

The effect of the pressure gradient on statistical properties poses thus a challenge far from being assessed. One pathway to obtain a better understanding of APG TBLs is based on the dynamics of the coherent structures. Large-scale features are indeed known to provide a significant contribution to the turbulent kinetic energy and Reynolds stress production in wall-bounded flows [21]. It is thus expectable that a better understanding of the pressure-gradient effects on the large-scale features in TBL flows will allow to improve the current turbulence models and flow control strategies. The influence of the pressure gradient can be observed in Ref. [22], which documents that attached-eddy-based models, which reproduce well ZPG TBLs statistical properties, fail when they are used to reconstruct the shear-stress distributions in the outer layer of APG TBLs. Consequently, it is concluded that large-scale motions in the outer layer have to be taken into account when modeling the turbulence production. Spectral and scale-decomposition analyzes [13] confirm that the large scales are more energized throughout the entire adverse-pressure-gradient boundary layer, especially in the outer region. Ref. [13] reports that the spectral distribution of energy in the wake region of APG TBLs is similar to that of the ZPG TBLs; nevertheless, the three-dimensional spatial correlations reported in Ref. [23] show that large-scale structures in the outer region of large-defect boundary layers are shorter in the streamwise direction and more inclined with respect to the wall.

In the present study, APG TBLs developing on a flat plate are experimentally studied in order to shed some light on the effect of the large-scale motions on the Reynolds stresses via combined analysis of statistics and modal decomposition. To this end, an experimental campaign was carried out by means of Particle Image Velocimetry (PIV) in a streamwise/wall-normal plane. Flow conditions were characterized in terms of the displacement-thickness-based Reynolds number $Re_{\delta ^{*}}$ and pressure-gradient parameter *β* by means of hot-wire anemometry (HWA) measurements performed in the Reynolds-number range $8000 < Re_{\delta ^{*}}< 34000$, and for pressure-gradient magnitudes of *β* = 1.3 and 2.4. Turbulence statistics were compared with Large-Eddy Simulation (LES) results of ZPG TBLs at similar Reynolds numbers [24] and LES from APG TBLs at comparable values of the Clauser pressure-gradient parameter *β* [2]. The effects of APGs on the large-scale structures are addressed with Proper Orthogonal Decomposition (POD) of the flow-fields.

The structure of the article is as follows: in Section 2 we report a description of the experimental setup, providing details on the streamwise evolution of *β*; in Section 2 we also assess the accuracies of different PIV approaches ranging from Ensemble Particle Tracking Velocimetry (EPTV) to single-pixel and standard PIV, using as a reference well-resolved hot-wire anemometry measurements. In Section 3 the discussion focuses on the comparison of flow statistics, taking into account also the effect of the streamwise evolution of *β*. Section 4 reports the modal decomposition of the flow allowing to assess the effect of *β* on the large-scale organization. Following an approach similar to Ref. [25], the instantaneous fluctuating velocities are decomposed into large-scale and small-scale features using POD modes in the streamwise/wall-normal planes.

## Experimental Setup

### Wind-tunnel and boundary-layer flow conditions

The experiments were performed in the *Minimum Turbulence Level* (MTL) closed-loop wind tunnel located at KTH Royal Institute of Technology in Stockholm. The test section is 7 m long with a cross-sectional area of 0.8 × 1.2 m^2^(height ×width). The MTL is capable of reaching a maximum speed of 70 m/s with a streamwise velocity fluctuation intensity of approximately 0.025% of the free-stream velocity at a test speed of 25 m/s. The air temperature can be controlled with an accuracy of ± 0.05 K by means of a heat exchanger. More details regarding the MTL can be found in Refs. [26, 27].

The desired streamwise evolution of the pressure gradient was established by means of wall inserts made of foam and hung by threaded rods. The roof shape could be further modified by adjusting the wind tunnel ceiling, which comprises a total of six panels allowing vertical displacement. The wall inserts were designed iteratively. The first trial shape of the ceiling was designed by performing Reynolds-Averaged Navier–Stokes (RANS) computations. From this geometry the final shape was iterated using as a reference the *β* distribution obtained from hot-wire measurements. As described in Ref. [9], the RANS computations were carried out by considering the two-equation Shear-Stress Transport (SST) model [28], implemented in the CFD code Fluent (v.6.3).

**Fig. 1:**
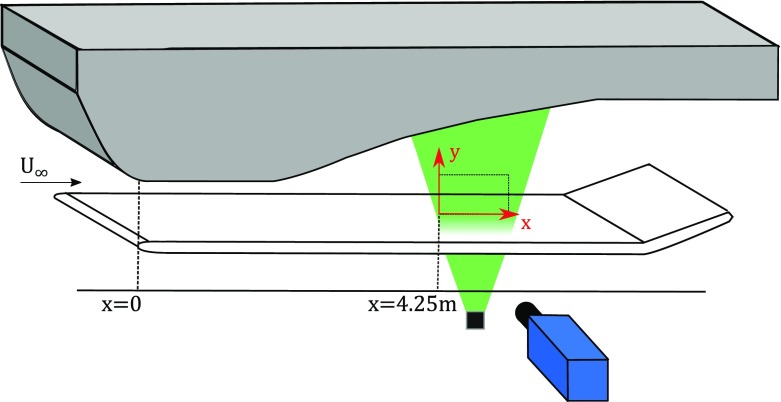
Description of the roof geometry and schematic view of the experimental setup. The wall insert to obtain the desired pressure-gradient evolution is indicated in gray. Note that both the upstream and downstream ends are flash-mounted with the tunnel roof

The turbulent boundary layers developed on a smooth aluminum flat plate of 6 m length and 26 mm thickness, spanning the entire width of the wind tunnel and suspended 15 cm above the wind-tunnel floor. The ceiling geometry was designed with a converging-diverging shape (as schematically shown in Fig. 1), thus resulting in an initially accelerated flow (i.e. a favorable pressure gradient), a region of nearly zero-pressure-gradient conditions and finally a region of adverse pressure gradient. The flow was initially accelerated by reducing the tunnel test section height from 0.80 m to approximately 0.60 m. The flat plate was placed at a vertical distance of 0.42 m from the roof at the throat. The leading edge of the flat plate was located right at the beginning of the roof throat. Downstream of the leading edge of the flat plate, the ceiling geometry was designed such that a ZPG was maintained for approximately 1.0 m. From that location on, two different adverse-pressure-gradient conditions were imposed by changing the roof geometry in the divergent part.

**Fig. 2:**
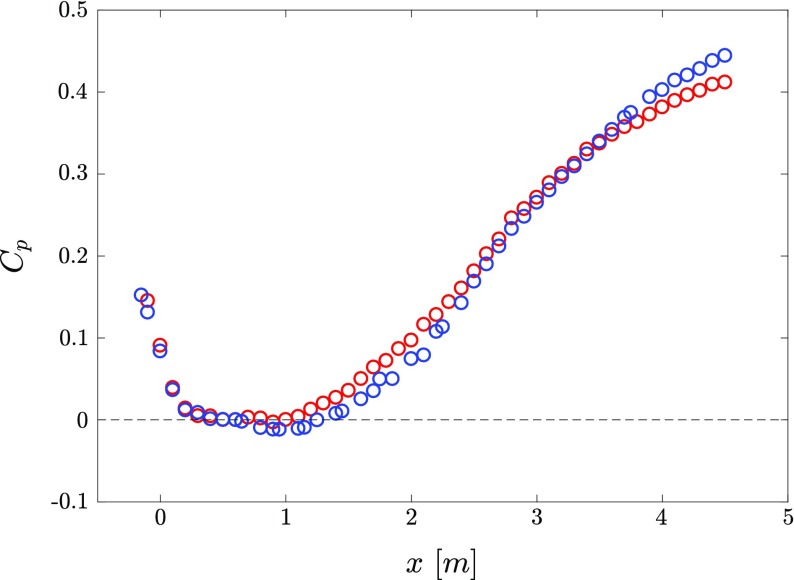
Distributions of pressure coefficient *C*_*p*_along the streamwise direction for two wall-insert configurations where () corresponds to the roof configuration 1 and () to the roof configuration 2. Note that the reference pressure for *C*_*p*_is taken at *x* = 0.6 m

The pressure distribution is expressed in terms of the pressure coefficient *C*_*p*_, which is defined for an incompressible flow as $C_{p}=(P-P_{ref})/(1/2\rho U^{2}_{ref})= 1-(U_{\infty }/U_{ref})^{2}$, where *P* is the local static pressure, *P*_*ref*_is the static pressure in the ZPG region (measured at *x* = 0.6 m), *U*_*∞*_ is the local free-stream velocity and *U*_*ref*_ is the reference free-stream velocity at *x* = 0.6 m. The experiments were carried out for three different inflow velocities, i.e. *U*_*ref*_ = 6, 12 and 30 m/s. The evolution of *C*_*p*_along the streamwise direction for the two aforementioned roof geometries is presented in Fig. 2.

The flat plate has a leading edge following the shape of a modified super ellipse and is equipped with a 1.5 m long trailing-edge flap in order to modify the position of the stagnation point. In the present experimental campaign, the flap position was set to 10^∘^. For a more detailed description the reader is referred to Ref. [27]. The boundary layer was tripped close to the leading edge with *DYMO* tapes (with the embossed letter ‘V’ pointing in the flow direction and a nominal height of 0.3 mm) in combination with a 1.6 mm height turbulator. Care was taken to ensure that the turbulent boundary layer at the measurement location was not affected by tripping effects [29]. The values of the Reynolds numbers and *β* for the various cases under consideration are reported in Table 1. The corresponding values of the shape factor *H*_12_ = *δ* ^∗^/*𝜃*(with *𝜃* being the momentum thickness) and of the viscous length *l* ^∗^ = *ν*/*u*_*τ*_ (with *ν* being the kinematic viscosity and *u*_*τ*_being the friction velocity) are also indicated for reference. In order to calculate *δ* ^∗^ and *𝜃*, the boundary-layer thickness needs to be determined, since it is the upper limit of integration. This quantity is rather ambiguous in APG TBLs owing to possible gradients of the streamwise velocity beyond the boundary-layer edge [30]. In this work, *δ*_99_has been calculated according to the procedure reported in Ref. [30], which is based on the diagnostic-plot concept [31].

**Table 1:** Boundary-layer parameters of the various cases in the present experimental database

*β*	$U_{\infty }$	$Re_{\delta ^{*}}$	*R* *e*_*𝜃*_	*R* *e*_*τ*_	*δ*_99_	*H*_12_	*l* ^∗^	Symbol	Roof
[−]	[m/s]	[−]	[−]	[−]	[mm]	[−]	[*μ* m]	Color	Configuration
1.3	9.4	13940	9070	1920	98.9	1.54	51.6		1
1.3	24.1	29950	20450	4130	90.1	1.46	21.8		1
2.4	4.8	8640	5340	1070	108.4	1.62	101.1		2
2.4	9.1	15850	9790	1880	104.3	1.62	55.4		2
2.4	23.4	33770	22240	4200	95.6	1.52	22.8		2

**Fig. 3:**
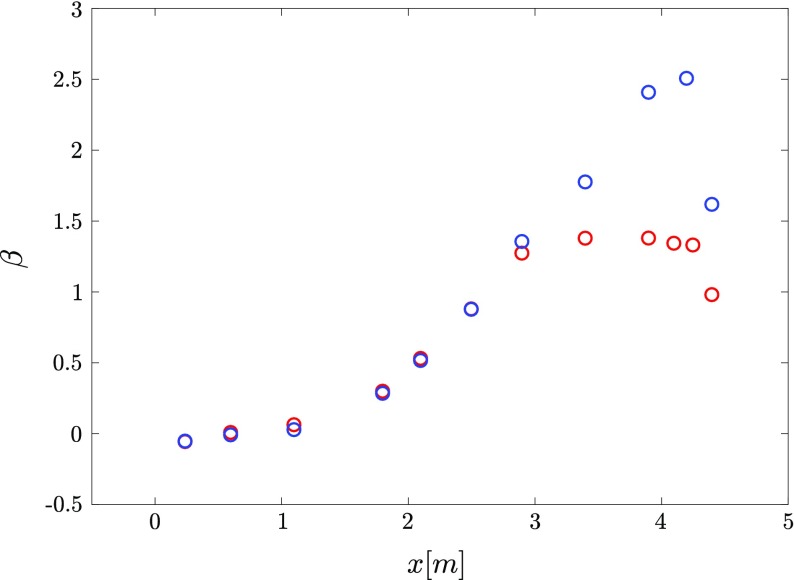
Streamwise distribution of the Clauser pressure-gradient parameter *β* where () corresponds to the roof configuration 1 and () to the roof configuration 2

Empirical evidence covering a wide range of Reynolds number and pressure-gradient parameters [30] has established that the classical boundary-layer edge corresponding to *U*/*U*_*∞*_ equal to 0.99 is found where $u^{\prime }/(U_{\infty }\sqrt {H_{12}})= 0.02$. This allows to calculate *U*_*∞*_ in an iterative way (since the shape factor *H*_12_is not known *a priori*), and once the value of *U*_*∞*_ is estimated, the value of *δ*_99_ can be obtained from the definition *δ*_99_ = *y*(*U* = 0.99*U*_*∞*_). The mean wall-shear stress has been deduced from hot-wire measurements in the sublayer (viscous sublayer and buffer region). The composite profile given by Ref. [32] is used to fit the experimental data up to *y* ^+^ = 15and correct the absolute wall position. The resulting distributions of *β* along the streamwise direction are reported in Fig. 3 for both roof configuration 1 and 2. Note that the *β* distributions reported in Fig. 3 are relative to *R* *e*_*τ*_ = 1920and 1880, respectively; however, the streamwise evolution of *β* has shown very weak dependence on *U*_*ref*_.

### Particle image velocimetry

Particle Image Velocimetry was used to perform velocity field measurements in a streamwise/wall-normal plane at a streamwise location of *x* = 4.25 m from the leading edge of the flat plate. In order to enable laser illumination for the PIV measurements an aluminum section of the flat plate was replaced with a transparent acrylic glass (polymethyl metacrilate) insert. The flow was seeded with 1 *μ* m diameter Di-Ethyl-Hexyl-Sebacate (DEHS) droplets produced with a smoke generator. Seeding particles were injected into the flow at the end of the test section to minimize flow perturbation and were then recirculated through the wind tunnel. The seeded flow was illuminated by a Quanta Ray double cavity Nd:YAG laser with a pulse energy of 400 mJ at 15 Hz. Laser light passed through the transparent section of the flat plate. The thickness of the laser sheet was approximately 1 mm.

The acquisition of the PIV images was performed with an *ANDOR Zyla sCMOS 5.5MP* camera (2560 × 2160 pixel array, 6.5 *μ* m × 6.5 *μ* m pixel size). The camera was equipped with a Tokina 100 mm lens. The lens aperture was set to *f*/*#* = 11and the objective was slightly set out of focus in order to obtain large particle images and avoid peak locking. The field of view was designed to fit the entire boundary-layer thickness with a spatial resolution of 16 pixels/mm. With the provided optical setup, the diffraction-limited particle image diameter was 15.7 *μ* m, and the depth of field was 38 mm. An ensemble of 1150 image couples was acquired for each experiment. Image quality was improved by removing laser reflections and illumination background using the POD-based approach described in Ref. [33].

A custom made PIV software developed at University of Naples Federico II was used to perform digital cross-correlation analysis of the particle images [34] to calculate the velocity fields. The interrogation strategy is an iterative multi-grid/multi-pass [35] image deformation algorithm [36], with final interrogation windows of 40 ×40 pixels with 75% overlap (the final vector spacing is equal to 10 pixels, i.e. 0.6 mm, which results in at least 140 vectors throughout the boundary layer thickness). B-spline interpolation schemes were used to improve the accuracy of the PIV processing [37]. The vector validation to identify invalid vectors was carried out with a universal median test [38] on a 3 × 3 vectors kernel and an error threshold equal to 2. Discarded vectors were replaced with a distance-weighted average of neighboring valid vectors.

### Hot-wire anemometry measurements

Hot-wire anemometry measurements were carried out to assess the quality of the PIV data and to characterize the pressure distribution along the streamwise direction. The measurements were performed by means of a home-made single hot-wire probe resembling a standard *Dantec* boundary-layer probe, i.e., a 55P15. The hot-wire probe consists of a fully-etched Platinum wire of 525 *μ* m length and nominal diameter of 2.5 *μ* m, which was soldered to conical prongs with diameters of around 30 *μ* m. Voltage signals from the hot-wire were recorded using a *Dantec StreamLine* 90N10 frame in conjunction with a 90C10 constant-temperature anemometer module operated at a resistance overheat ratio of 80%. An offset and gain were applied to the top-of-the-bridge voltage in order to match the voltage range of the 16-bit A/D converter. A low-pass filter of 30 kHz cut-off frequency was used prior to the data acquisition in order to avoid aliasing. The calibration of the hot-wire was performed in-situ using as reference a Prandtl tube located parallel to the incoming freestream. The Prandtl tube was connected to a micromanometer of type *FC0510 (Furness Control Limited)*, which was also employed to record the ambient temperature and pressure during the calibration and the experiments. Data acquired in the calibration was fitted to a fourth-order polynomial curve [39]. The uncertainty of hot-wire measured mean velocity and turbulence intensity is estimated to be 1% and 2%, respectively.

Hot-wire measurements were acquired with a sufficiently large number of points within the viscous sublayer and the buffer region in order to correct for the absolute wall position and determine the friction velocity [40] without relying on log-law constants.

### Spatial resolution effects of PIV on turbulence statistics

The turbulence statistics evaluated with PIV can be affected by limited spatial resolution issues due to the finite size of the interrogation window [41–44]. This induces systematic errors on the mean velocity in the presence of a mean velocity gradient. The second-order statistics are similarly affected by limited resolution issues, since small-scale features are filtered out in the PIV processing, and so is their energy content. On the other hand, the effect of random noise on the shape of the cross-correlation peaks is to produce a white noise distribution over the whole spectrum [45], which under certain conditions might fictitiously compensate the previous modulation effect [46].

In this section two different approaches to improve the spatial resolution and accuracy of turbulence statistics are compared:

- ◦ Ensemble-correlation using the probability density function (pdf) estimation approach outlined in Ref. [47] to extract second-order statistics. The interrogation region size was equal to 41 × 11pixels. A symmetric double-correlation method [48] was used to improve convergence. Additionally, the correlation maps were spatially averaged over a region of 400 × 4pixels (in the streamwise and wall-normal directions).
- ◦ Ensemble Particle Tracking Velocimetry (EPTV), as in Refs. [49–51], with biased search using PIV as a predictor [52]. The bin is performed on 400 × 4pixels regions. The computation of turbulence statistics is carried out with a standard *top-hat* bin averaging and with a polynomial-fit-based method [49], which estimates the statistical moments around a second-order polynomial fit applied on the velocity vectors within each bin. This method is here assessed for the first time in wall-bounded flows, and it has demonstrated in shear-free flows to reduce systematic errors due to unresolved mean velocity gradients.

**Fig. 4:**
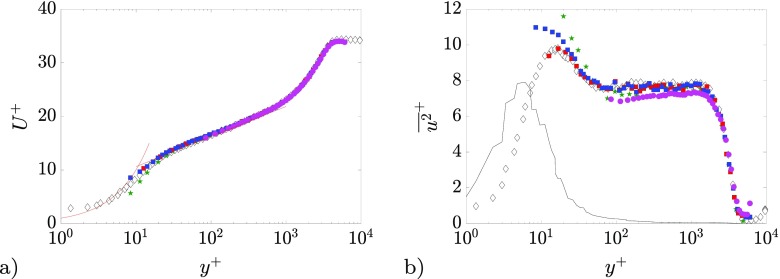
Comparison between HWA (◇), standard-PIV (), *ensemble correlation* (), ensemble Particle Tracking Velocimetry with top-hat () and polynomial fit approach () for the case with *R* *e*_*τ*_ = 4130and *β* = 1.3. **a** Mean streamwise velocity in inner scaling and **b** streamwise normal Reynolds stress in inner scaling. Additionally, red lines () depict the linear profile *U* ^+^ = *y* ^+^and the logarithmic profile $U^{+}=\tfrac {y^{+}}{0.41}+ 5$ in a) and the black line (–) depicts the wall-normal velocity gradient 10 ⋅ *∂* *U* ^+^/*∂* *y* ^+^profile in **b**)

The accuracy of the various approaches is tested on a case with *β* = 1.3 and *R* *e*_*τ*_ = 4130; the results are presented in Fig. 4. Hot-wire measurements are included for reference. No spatial resolution effects are expected in the overlap region since the viscous-scaled wire length of the hot-wire, defined as *L* ^+^ = *L*/*l* ^∗^ (where *L* is the active hot-wire length), is *L* ^+^ ≈ 24[53, 54]. The PIV results reported in Fig. 4 are in good agreement with the mean velocity profile measured by means of hot-wire anemometry from the wake region down to the overlap region (*y* ^+^ ≈ 100). The inner-scaled streamwise variance profile $\overline {u^{2}}^{+}$ exhibits an intensity reduction of about 10% from *y* ^+^ ≈ 100 to *y* ^+^ ≈ 2000. Nevertheless, the shape of the profile is correctly estimated if compared with the hot-wire profile. The attenuation is thus to be ascribed to modulation of the small-scale fluctuations. Nonetheless, considering that the modulation appears to be almost independent of the wall-normal position, it can be hypothesized that the spectral content of energy of the small scales exhibits small changes for 100 ≤ *y* ^+^ ≤ 2000 if compared to the large-scales contribution (as in Figure 6 of Ref. [3]). Thus, the energy spatial distribution obtained from PIV data is only weakly affected by non-uniform modulation effects.

The ensemble-correlation and ensemble-PTV methods lead to a very good agreement with the reference mean velocity profile from hot-wire measurements down to approximately *y* ^+^ = 10. It has to be underlined here that the case under analysis is one of the two cases with higher *R* *e*_*τ*_, and thus one of the most challenging of the dataset from the standpoint of the spatial resolution. Within the inner layer the ensemble-correlation approach is biased toward smaller velocity values than the reference profile measured by the hot-wire. This bias can be attributed to the residual reflections present on the images after pre-processing, which affect the computed correlation maps by stretching them along the wall-parallel direction, as well as biasing their peak toward zero-displacement. A thorough assessment of bias errors in ensemble correlation near walls is reported in Ref. [55], in which it is suggested to use ensemble correlation or PTV for wall distances below half the PIV interrogation window size, while PTV is superior for wall distances smaller than the particle image diameter. Both ensemble-correlation and ensemble-PTV measurements of the $\overline {u^{2}}^{+}$ are in good agreement with the hot-wire data in the outer layer. The ensemble-correlation approach overestimates $\overline {u^{2}}^{+}$ for *y* ^+^ < 100. Similarly to the bias in the mean velocity profile, this error can be attributed to the stretching of the correlation peak along the wall-parallel direction due to the residual reflections on the pre-processed images. The ensemble-PTV approach is able to follow the reference profile well within the inner layer with a remarkable improvement when using the polynomial fit approach [49]. Indeed, in the regions where *∂* *U* ^+^/*∂* *y* ^+^attains its larger values the residual unresolved velocity gradient within the interrogation window might lead to significant overestimation of the normal Reynolds stresses [49].

On the basis of this assessment, in the following sections statistics obtained exclusively using the ensemble-PTV approach with polynomial fit [49] will be shown. The data obtained from standard-PIV will be used only for the purpose of analyzing the large-scale flow-field organization using Proper Orthogonal Decomposition, as discussed in Section 4.

## Turbulence Statistics

**Fig. 5:**
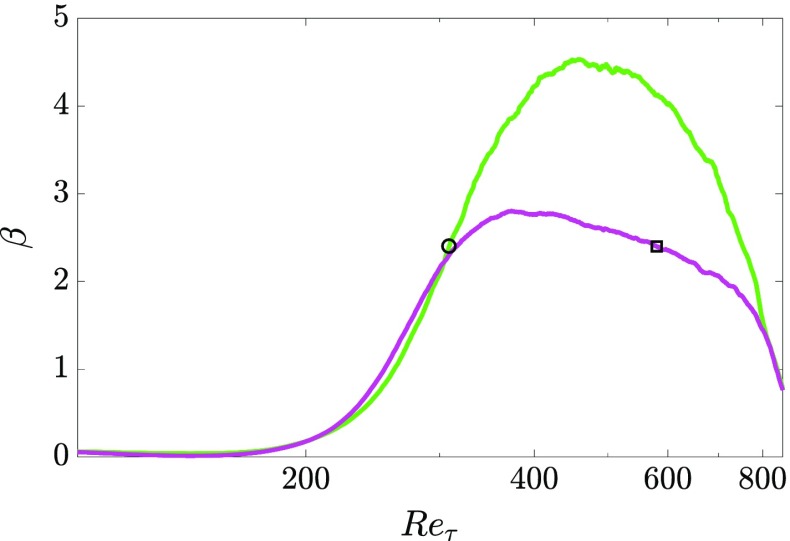
Streamwise distributions of the Clauser pressure-gradient parameter *β* for the two APG LES cases. Colors are reported in Table 2. Circle symbol (∘) indicates conditions of the increasing-*β* case and square symbol ($\square $) indicates those of the decreasing-*β* case, as reported in Table 2

In this section the influence of *β* and *R* *e* on the turbulence statistics is addressed. The main focus is on the effect of different local *β* values and streamwise evolutions of *β* on first- and second-order statistics while the discussion of the impact on the flow organization is postponed to Section 4. Data from well-resolved LESs of a ZPG TBL [24] and APG TBLs [2, 56] are included to further support the discussion. The simulation parameters for these cases are reported in Table 2. The evolution of *β* in the two APG LES cases as a function of *R* *e*_*τ*_is reported in Fig. 5.

**Table 2:** Boundary-layer parameters for the LES dataset

*β*	$Re_{\delta ^{*}}$	*R* *e*_*𝜃*_	*R* *e*_*τ*_	*H*_12_	Case	Reference	Symbol/Color
0	8705	6380	1940	1.36	ZPG	[24]	
2.4	2290	1330	315	1.72	increasing *β*	[2, 56]	
2.4	5130	2930	580	1.75	decreasing *β*	[2, 56]	

### Effect of *β* at matched Reynolds number

**Fig. 6:**
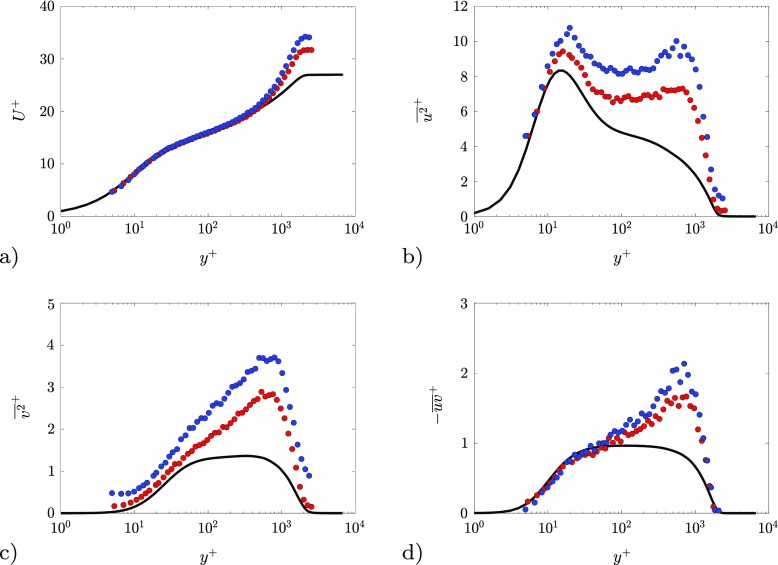
Inner-scaled profiles for a ZPG (−), and APG TBLs with *β* = 1.3() and *β* = 2.4() at *R* *e*_*τ*_ ≃ 1900. **a** Mean streamwise velocity, **b** streamwise normal Reynolds stress, **c** wall-normal Reynolds stress and **d** Reynolds shear stress

In Fig. 6 a comparison between inner-scaled turbulence statistics for *β* = 1.3 () and *β* = 2.4 () at matched friction Reynolds number *R* *e*_*τ*_ ≈ 1900is reported. At this point a comment on the choice of the definition of the Reynolds number is appropriate. In the case of strongly-decelerated APG TBLs, the friction velocity (and hence the friction Reynolds number *R* *e*_*τ*_) would approach zero, and would therefore be inappropriate to define the state of the boundary layer. However, for the present rather mild pressure-gradient conditions, both *R* *e*_*𝜃*_ and $Re_{\delta ^{*}}$ develop similarly to *R* *e*_*τ*_[2, 57]; thus, *R* *e*_*τ*_is an appropriate Reynolds number to study the *R* *e*-dependence of the cases analyzed here. As a baseline for comparison, Fig. 6 also reports velocity and Reynolds-stress profiles from a ZPG TBL simulation at matched *R* *e*_*τ*_ ≈ 1900 [24]. Doing so, the effect of the imposed pressure gradient can be assessed. The APG mean velocity profiles collapse with the ZPG profile from the wall up to *y* ^+^ ≈ 200, thus showing no significant discrepancy with the law of the wall. A more prominent wake is observed in APG TBLs when comparing with the ZPG case. This is due to the reduced wall-shear stress present in APGs, which is connected to the increased wall-normal convection. The increase of momentum defect in the wake, reported for instance in Refs. [3, 4, 8, 13, 20], strongly depends on the flow history and accumulated effect of the APG, as discussed in Ref. [2].

The Reynolds normal-stress profiles in the APG TBLs exhibit an outer peak located at around 500 ≤ *y* ^+^ ≤ 700, which is not present in the ZPG case. The amplitude of the outer peak increases with *β*. As addressed in Ref. [13], the increase of the inner-scaled Reynolds stresses is not just due to the lower value of the friction velocity used to scale the profile, but it is ascribed to enhanced large-scale motions in the outer region. This is further supported by the distribution of the Reynolds shear stress $-\overline {uv}^{+}$, which plays a leading role in the turbulence production, as discussed in Section 3.3. It is worth noting that the inner-scaled edge velocity increases with *β*, a fact that is connected to the presence of the additional mean shear in the outer region due to the pressure gradient [20, 58].

### Effect of Reynolds number at matched *β*

**Fig. 7:**
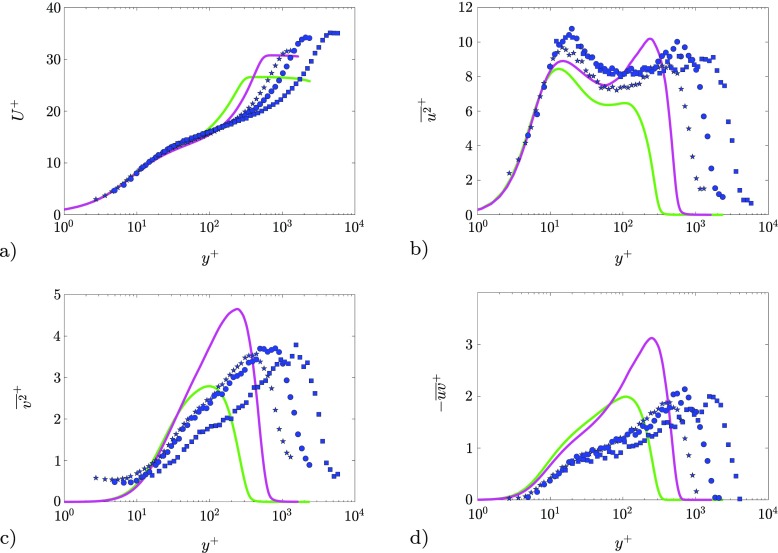
Inner-scaled profiles for *β* = 2.4at varying Reynolds number. PIV measurements are represented with symbols: () *R* *e*_*τ*_ = 1070, () *R* *e*_*τ*_ = 1880and () *R* *e*_*τ*_ = 4200. LES profiles are represented with solid lines: () *R* *e*_*τ*_ = 315and () *R* *e*_*τ*_ = 580. **a** Mean streamwise velocity, **b** streamwise normal Reynolds stress, **c** wall-normal Reynolds stress and **d** Reynolds shear stress

Turbulence statistics are compared at fixed *β* ≈ 2.4 for *R* *e*_*τ*_values of 1070, 1880 and 4200. The profiles are shown in inner scaling in Fig. 7. Statistics from LES of APG TBL at matched local *β* [2, 56] at *R* *e*_*τ*_values of 315 and 580 are also included for comparison. Note that, in the cases under consideration, although the local value of *β* is matched, the *β*(*x*)evolution is not the same for all cases (see Figs. 3 and 5). The streamwise velocity variance profiles for the experimental cases are characterized by inner and outer peaks with limited intensity variation in the investigated Reynolds-number range. This is in agreement with Ref. [59], which reported that the large-scale contribution increases weakly with the Reynolds number. The Reynolds-number range under investigation is, however, not large enough to draw firm conclusions about the amplitude of the outer peak documented in Ref. [60].

Considering the relatively weak influence of the Reynolds number at matched *β* on the magnitude of inner/outer peaks, the differences observed between LES and experimental data can be attributed mostly to the different flow histories present in the various cases (see Figs. 3 and 5). For the case of decreasing *β*, all the Reynolds stresses have a larger outer peak, as a result of a stronger accumulated *β* history experienced throughout its development (see Ref. [2]). Moreover, as reported in Ref. [61], low-Re TBLs are more sensitive to APG effects, especially when it comes to the development of energetic structures in the outer region of the boundary layer. This therefore justifies the stronger outer peak in the low-Re TBL. Similarly, the LES case with increasing *β* shows an attenuated outer peak in the $\overline {u^{2}}^{+}$ profile, due to reminiscence of lower pressure-gradients. The LES and experimental data at matched local *β* support the conclusion that the outer-layer features are strongly dependent on the streamwise evolution of *β*, thus hindering a comparison of APG TBLs at matched Reynolds number and *β* if the upstream history is not known, as it is often the case in several (comparative) studies in the literature.

### Turbulence production

Further insight on the effect of the APG on the large-scale dynamics can be obtained via analysis of the turbulence production. The general equation for turbulence production in inner scaling, assuming a mean spanwise velocity of zero [62], can be written as follows:

1 $$ P^{+}=-\overline{uv}^{+}\frac{\partial U^{+}}{\partial y^{+}}-\left( \overline{u^{2}}^{+}-\overline{v^{2}}^{+}\right)\frac{\partial U_{\infty}^{+}}{\partial x^{+}}-\overline{uv}^{+}\frac{\partial V^{+}}{\partial x^{+}}.  $$

Through an order-of-magnitude analysis it can be shown that in ZPG or in mild pressure-gradient TBLs the second and third terms of the right-hand side of Eq. 1 are negligible with respect to the first one [13]. This allows for a simplified estimation of the turbulence production as:

2 $$ P^{+}\approx-\overline{uv}^{+}\frac{\partial U^{+}}{\partial y^{+}}. $$

**Fig. 8:**
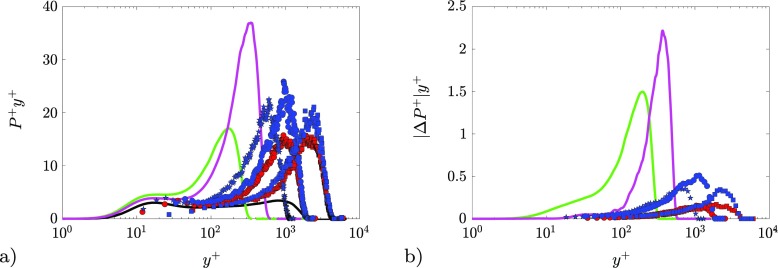
Premultiplied turbulence production in inner scaling. Colors and symbols are reported in Tables 1 and 2: **a** turbulence production estimated according to Eq. 1 and **b** error in the estimation of the turbulent production due to Eq. 2. Note that the ordinate in **b**) is zoomed in to enhance the minor differences

The inner-scaled turbulence production in premultiplied form (calculated from Eq. 1) is reported in Fig. 8a for all the cases under study. The visual advantage of the premultiplied form *P* ^+^ *y* ^+^is that, when represented in semi-logarithmic form, equal areas correspond to equal contributions to the production [1]. While the ZPG TBL is characterized by a relatively flat *P* ^+^ *y* ^+^distribution, in the case of the APG TBL an increasing production is observed in the outer layer, in agreement with Refs. [13, 63]. This depicts a scenario of increasingly more energetic large-scale motions in the outer layer [13]. Interestingly, the position of the production peak corresponds to the location of the peak in the $\overline {uv}$ profile and is weakly dependent on *β* at fixed *R* *e*_*τ*_when scaled in inner units. This means that the main effect of the pressure gradient is to change the distribution of energy through the boundary layer, displacing large energetic structures from the near-wall region to the outer region (as it will be further highlighted in Section 4). This originates from the fact that the scale separation is fixed when considering TBLs at matched *R* *e*_*τ*_. The APG thickens the boundary layer and convects flow in the wall-normal direction, but, when carrying out the comparisons at fixed *R* *e*_*τ*_, the outer peak is at approximately the same location.

The effect of *R* *e*_*τ*_is to shift the production peak toward higher *y* ^+^. LES data again confirm the importance of history effects: even though both cases are for *β* ≈ 2.4, the producion peak intensity for the case of increasing *β* is similar to the experimental case at *β* = 1.3. Conversely, for the case with decreasing *β*, the observed production peak intensity would be compatible with a case with constant *β* higher than 2.4.

As stated above, the use of Eq. 2 results in an approximation to *P* ^+^, and the difference between the production obtained from Eqs. 1 and 2 is denoted by ${\Delta }_{P^{+}}$. Inner-scaled profiles of ${\Delta }_{P^{+}}$ in premultiplied form are reported in Fig. 8b. Since the turbulence production peaks are located at the same position as those of $\overline {uv}^{+}$, the maximum error in the estimation of the turbulent production (due to the use of Eq. 2) is located at the position where the maximum values of *P* ^+^ *y* ^+^ are observed in Fig. 8a). Note that the third term on the right-hand side in Eq. 2, which involves $\overline {uv}^{+}$, becomes progressively larger with increasing values of *β*. The maximum values of ${\Delta }_{P^{+}} y^{+}$ reach around 6*%* and 10*%* of the maxima in *P* ^+^ *y* ^+^ in the two LES cases. This is reduced to around 2*%* in the higher-*R* *e*, experimental cases. Therefore, the approximation incurred in Eq. 2 appears to be reliable at moderately-high Reynolds numbers, and with mild pressure-gradient magnitudes.

## POD and Modal Contribution to Turbulence Statistics

In this section a modal analysis by means of Proper Orthogonal Decomposition [64] is carried out using data from standard PIV analysis. The effect of *β* and of the Reynolds number on the modal energy distribution and on the topology of the spatial modes is investigated and discussed. The modes are used to perform a low-rank approximation of the flow fields and to address the relative importance of large and small scales on the turbulence statistics.

### POD fundamentals

Consider the streamwise wall-parallel and crosswise wall-normal fluctuating velocity components, both functions of space $\underline {x}=(x,y)$ and time *t*, $u(\underline {x},t)$ and $v(\underline {x},t)$. These quantities can be approximated as a linear combination of basis functions $\phi _{n}(\underline {x})$ as:

3 $$ u(\underline{x},t) \approx \sum\limits_{n = 1}^{N_{m}}a_{n}(t)\phi_{n}(\underline{x}),  $$

where *a*_*n*_(*t*) are time-dependent coefficients. Note that an equivalent expression can be written for *v*. The symbol *N*_*m*_is used to indicate the number of basis functions used. In the limit *N*_*m*_ →*∞* the approximation becomes exact. Proper Orthogonal Decomposition identifies orthonormal basis functions, i.e. the scalar product between whichever pair of functions of the set is $\left (\phi _{n}(\underline {x}),\phi _{p}(\underline {x})\right )=\delta _{np}$, with *δ*_*np*_ being the Kronecker delta (equal to 1 for *n* = *p* and to 0 elsewhere). POD can be used to extract information regarding the coherent structures in turbulent flows since it sorts the spatial basis functions $\phi _{n}(\underline {x})$ according to its mean square projections $\lambda _{n}=\left \langle \left (u(\underline {x},t),a_{n}(t)\phi _{n}(\underline {x})\right )\right \rangle $, with 〈...〉 indicating an ensemble average. The identification of the basis functions corresponds to the solution of the integral eigenvalue problem having with kernel the two-point correlation tensor of *u*, with *λ*_*n*_ being the eigenvalues and $\phi _{n}(\underline {x})$ being the eigenvectors.

Consider a set of *N*_*t*_ realizations, each one consisting of *N*_*p*_ values along the spatial coordinate $\underline {x}$, with *N*_*t*_ < *N*_*p*_. The integral equation has a discrete set of solutions: *N*_*t*_ eigenvalues *λ*_*n*_ of the two-point correlation matrix and *N*_*t*_basis functions $\phi _{n}(\underline {x})$. Following the snapshot method [65], each realization can be treated as a *N*_*p*_-dimensional vector and the data can be arranged in a *N*_*t*_ × *N*_*p*_snapshot matrix:

4 $$ \underline{\underline{u}} = \left[ \begin{array}{ccccc} u(x_{1},t_{1}) & {\cdots} & u(x_{N_{p}},t_{1}) \\ {\vdots} & {\ddots} & {\vdots} \\ u(x_{1},t_{N_{t}}) & {\cdots} & u(x_{N_{p}},t_{N_{t}}) \\ \end{array} \right]; \ \underline{\underline{v}} = \left[ \begin{array}{ccccc} v(x_{1},t_{1}) & {\cdots} & v(x_{N_{p}},t_{1}) \\ {\vdots} & {\ddots} & {\vdots} \\ v(x_{1},t_{N_{t}}) & {\cdots} & v(x_{N_{p}},t_{N_{t}}) \\ \end{array} \right].  $$

Since the focus of this analysis is on the Reynolds stresses, it is suitable to extract a basis which is optimal in terms of turbulent kinetic energy, i.e. which maximizes both the *u* and *v* energy content. It is important to underline that, since planar PIV only provides two components of the velocity field, the analysis here is limited to the turbulent kinetic energy associated to streamwise and wall-normal velocity fluctuations. From this point on this would be referred as in-plane turbulent kinetic energy or simply TKE. The two-point correlation matrix can be written as $\underline {\underline {C}}=\underline {\underline {u}} \ \underline {\underline {u}}^{T}+\underline {\underline {v}} \ \underline {\underline {v}}^{T}$, where the superscript ^*T*^refers to the matrix transpose. Solving the eigenvalue problem of $\underline {\underline {C}}$ returns the eigenvalues *λ*_*n*_ and the left and right eigenvector matrices. The left and right eigenvector matrices are respectively the matrix $\underline {\underline {\psi }}$ containing in its columns the normalized temporal modes $\underline {a_{n}}/|\underline {a_{n}}|$ (which are orthonormal vectors of length *N*_*t*_and unitary norm) and its inverse (i.e. its transpose). Note that the columns of $\underline {\underline {\psi }}$ form a basis of rank *N*_*t*_ and that the eigenvalues *λ*_*n*_are representative of the in-plane turbulent kinetic energy contribution of each mode. The orthonormal spatial modes $\phi _{n}(\underline {x})$ can then easily be computed as $\underline {\underline {{\Sigma }_{u}}}\ \underline {\underline {\phi _{u}}}=\underline {\underline {\psi }}^{T}\ \underline {\underline {u}}$ and $\underline {\underline {{\Sigma }_{v}}}\ \underline {\underline {\phi _{v}}}=\underline {\underline {\psi }}^{T}\ \underline {\underline {v}}$ where $\underline {\underline {{\Sigma }_{u}}}$ and $\underline {\underline {{\Sigma }_{v}}}$ are diagonal matrix which in each *n* ^*th*^diagonal elements contain the streamwise and wall-normal Reynolds-stress contribution of the *n* ^*th*^mode.

### POD modes

**Fig. 9:**
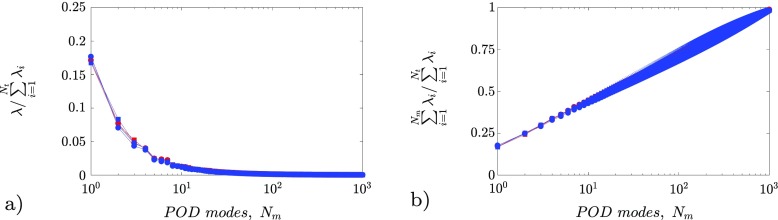
**a** POD spectrum of the eigenvalues *λ*_*i*_; **b** Cumulative sum of the eigenvalues. Colors and symbols are reported in Table 1

The eigenspectral distribution of energy of the POD modes is reported in Fig. 9. The eigenvalues distributions from the entire PIV dataset are superposed. The modal energy content is normalized with the corresponding total energy content $\sum \limits _{i = 1}^{N_{t}}\lambda _{i} $ of each case. Interestingly, the mode energy distribution is not appreciably affected neither by the Reynolds number nor by the pressure gradient magnitude. In particular, the mode energy distribution is in good agreement with the ZPG data presented in Ref. [66] at *R* *e*_*𝜃*_ = 8200, suggesting that in the considered range the energy share between large-scale and small-scale features is independent of both *R* *e* and *β*. About 20% of the energy contribution is ascribed to the first mode, 10% is ascribed to the second mode and barely 5% to the third and the fourth modes. It is thus possible to model up to 40% of the in-plane turbulent kinetic energy with only four modes, as shown in Fig. 9b. Consequently these modes, which are related to the large-scale motions, are discussed in detail in the following.

It has to be underlined here that the first POD modes relate to the large-scale motions, which populate the outer part of the boundary layer. Such motions scale with the outer length scale and typically have a streamwise length much larger than the measurement domain assessed in the current experiment. Consequently, the streamwise length of these large-scale motions cannot be fully characterized. Recent studies aiming at the characterization of streamwise extent of outer-scale features have exploited multiple cameras [67] or temporal resolution [68]. Nonetheless, snapshot POD allows to estimate the characteristic shape of these structures in the observed measurement domain since it is purely based on the two-point temporal correlation. In the following, the POD modes are compared with those reported in Ref. [66] for a ZPG TBL, which were obtained with a flow domain with similar size in outer scaling.

**Fig. 10:**
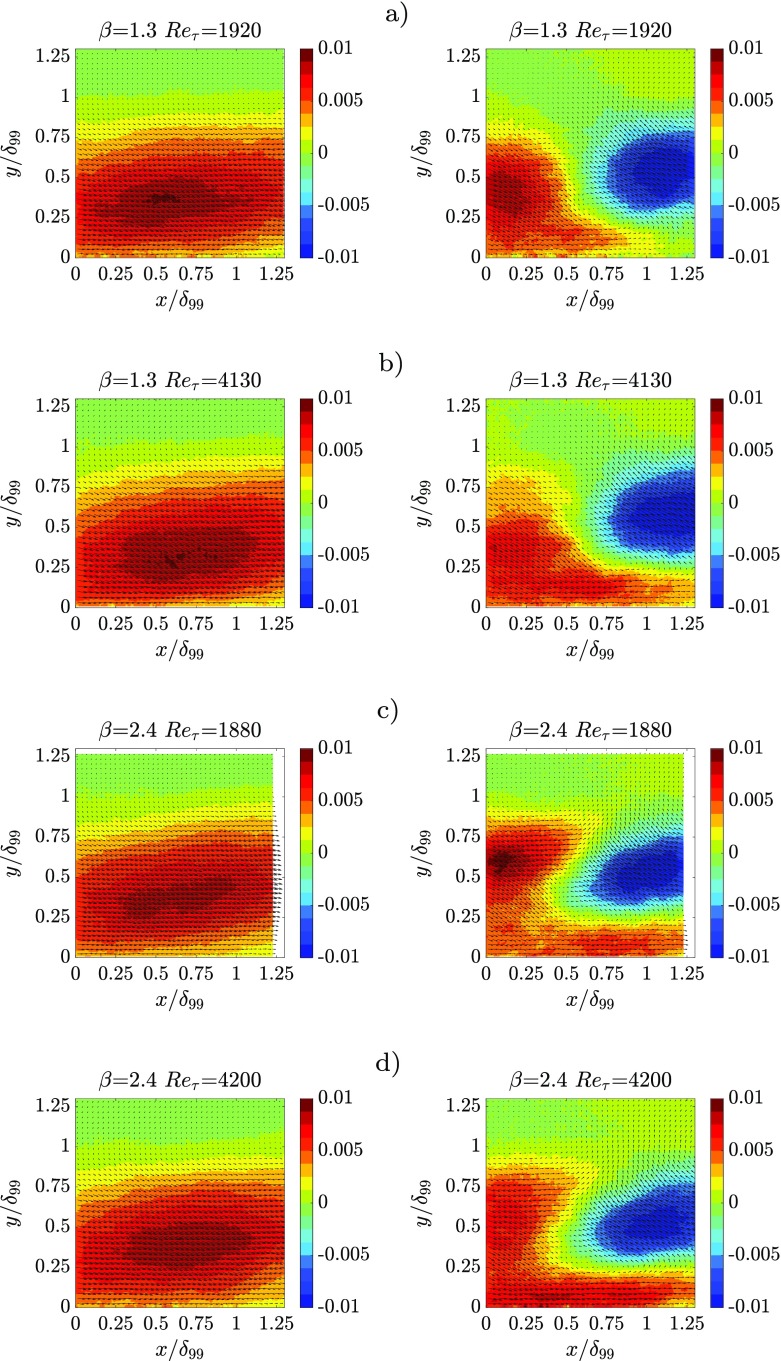
Contour plot with superimposed vector arrows of POD spatial modes *ϕ*_1_(left column) and *ϕ*_3_(right column) of the streamwise velocity fluctuations for: **a** *β* = 1.3and *R* *e*_*τ*_ = 1920, **b** *β* = 1.3and *R* *e*_*τ*_ = 4130, **c** *β* = 2.4and *R* *e*_*τ*_ = 1880, **d** *β* = 2.4and *R* *e*_*τ*_ = 4200

**Fig. 11:**
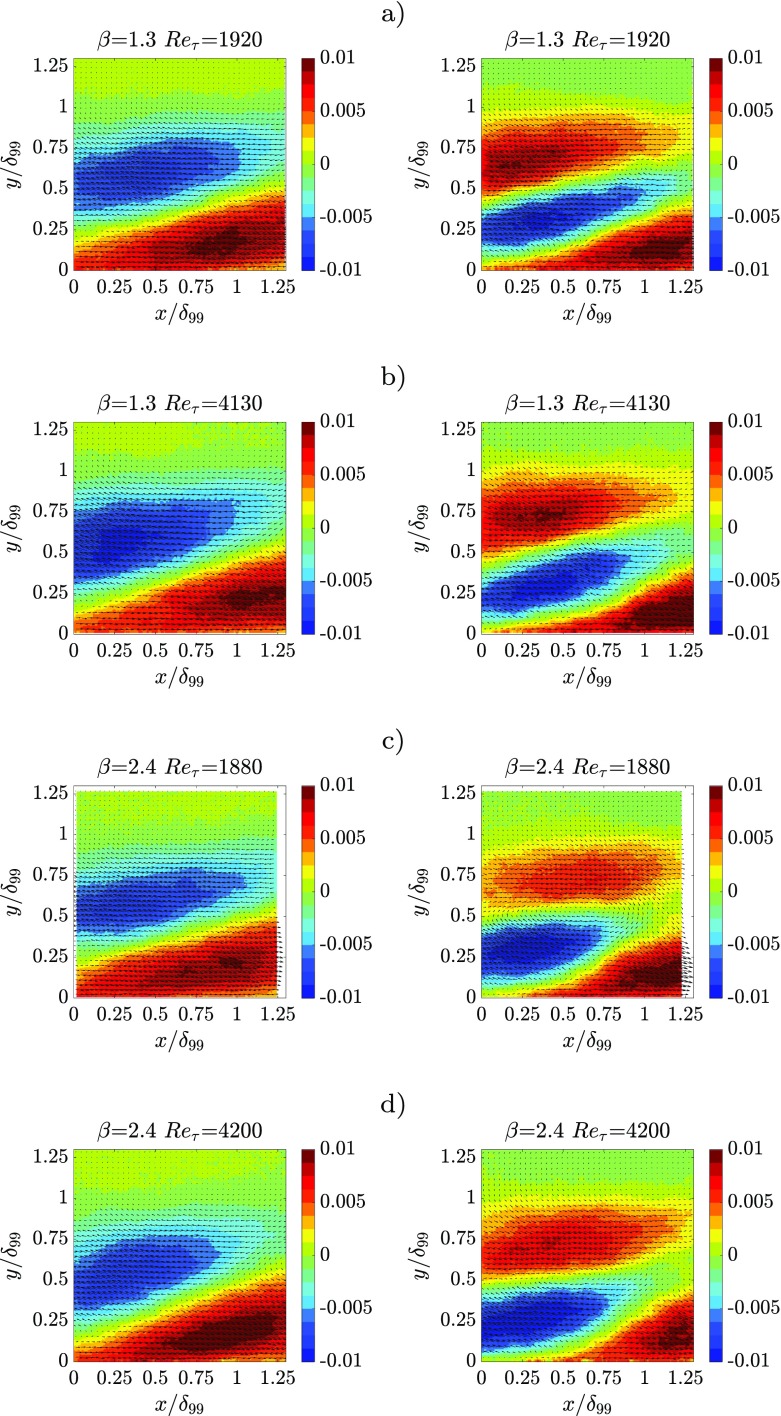
Contour plot with superimposed vector arrows of POD spatial modes *ϕ*_2_(left column) and *ϕ*_4_(right column) of the streamwise velocity fluctuations for: **a** *β* = 1.3and *R* *e*_*τ*_ = 1920, **b** *β* = 1.3and *R* *e*_*τ*_ = 4130, **c** *β* = 2.4and *R* *e*_*τ*_ = 1880, **d** *β* = 2.4and *R* *e*_*τ*_ = 4200

The streamwise velocity contours and vector fields of the spatial modes are plotted for *β* ≈ 1.3 and 2.4 and *R* *e*_*τ*_ ≈ 1900 and 4200 in Fig. 10 (which reports modes 1 and 3) and in Fig. 11 (modes 2 and 4). This particular choice is due to the similar spatial organization of these mode pairs in all tested cases. The spatial coordinates are scaled using the boundary-layer thickness *δ*_99_. The first spatial mode represents an event with positive streamwise velocity and negative wall-normal velocity. According to the quadrant analysis reported for instance in Ref. [69], such an event is a “sweep”, and is denoted as a Q4 event which brings high-momentum flow toward the wall. It has to be noted, however, that fluctuating instantaneous flow fields are obtained as a linear combination of the spatial modes, each one multiplied by their respective time coefficient as in Eq. 3: if multiplied by a negative time coefficient, the first spatial mode will instead represent a Q2 event (*u* < 0, *v* > 0), i.e. an ejection. This result appears to confirm the result in Ref. [70] that sweeps and ejections should be essentially mirror images of one another. The shape of the first mode is similar to that shown in Ref. [66] for a ZPG TBL. By increasing *β*, however, the location of the region affected by more intense streamwise velocity fluctuations (i.e. sweeps/ejections) is moved farther from the wall. In Ref. [66] the streamwise velocity is reported to be stronger below *y* = 0.6*δ*_99_ while here it is found to extend well beyond *y* = 0.75*δ*_99_and *y* = 0.8*δ*_99_for *β* = 1.3and 2.4, respectively. This finding confirms the claim in Ref. [23] according to which, in APG TBLs, wall-attached large sweeps and ejections are less numerous than in ZPG TBLs.

Modes 1 and 3 (Fig. 10) are coupled: mode 3 represents a phase-quadrature term which is needed to correctly represent the low/high momentum coherent motions being convected inside or outside of the measurement domain. As shown by mode 3, the passage of high/low-momentum coherent motions results also in promoting high/low-momentum streaks close to the wall; this shows a connection between the outer-layer fluctuations and the near-wall dynamics. The effect of the Reynolds number is similar to the *β* effect since the mode spatial distribution is slightly changed with an increased outer fluctuation peak. It is also interesting to note (see mode 3, Fig. 10) that the increase of *β* intensifies the near-wall velocity fluctuations connected with the passage of the low/high momentum coherent motions. Modes 2 and 4 are reported in Fig. 11. Mode 2 represents a shear layer spanning through the boundary layer and going from *y*/*δ*_99_ ≈ 0.1 on the left of the domain to *y*/*δ*_99_ ≈ 0.5on the right of the domain. Although the inclination of the shear layer seems to slightly decrease with *β*, the investigated range is not large enough to draw firm conclusions. Note that the inclination is just slightly smaller than that reported in Ref. [66] for a ZPG TBL. Modes 2 and 4 seem to determine the location of the high/low-momentum coherent motions through the boundary layer. The effect of the Reynolds number is to increase the magnitude of the fluctuation maxima as for mode 1 and 3 and to move the maxima closer to the wall. The effect of *β* appears to be an overall increase in the penetration of sweeps/ejections from the outer layer toward the wall. Although the shear layer inclination is only weakly dependent on *β* and *R* *e*, the inclination of the coherent motions is changed, especially in presence of larger *β*, an observation that is particularly evident for mode 4.

In order to understand the modal contribution to the Reynolds stresses, a low-order reconstruction of rank *N*_*m*_of $\underline {\underline {u}}$ and $\underline {\underline {v}}$ is performed retaining the first *N*_*m*_modes in Eq. 3, as a counterpart of the results shown in Ref. [3], where hot-wire measurements were decomposed into small/large-wavelength contributions to observe the effect of the large-scale motions on $\overline {u^{2}}$. Here POD is used as a filter and allows to emphasize the role of the large-scale phenomena in the Reynolds stresses. For a discrete dataset as the present one, if the number of modes *N*_*m*_used for the low-order representation is equal to the number of realizations *N*_*t*_ the reconstruction is exact and accounts for the exact representation of the in-plane turbulent kinetic energy and Reynolds stresses. It has to be remarked that this reconstruction will be optimal for what concerns the in-plane turbulent kinetic energy, although we have checked that for the present problem it approximates satisfactorily the reconstruction of all the in-plane components of the Reynolds-stress tensor. A low-order representation allows also to separate scale contributions, as shown in Ref. [25], since large-scale features correspond to the higher-energy modes, while small scales are contained in the higher-order modes; thus, performing a low-rank reconstruction allows to separate and highlight the contribution of large-scale structures in building up the Reynolds stresses.

**Fig. 12:**
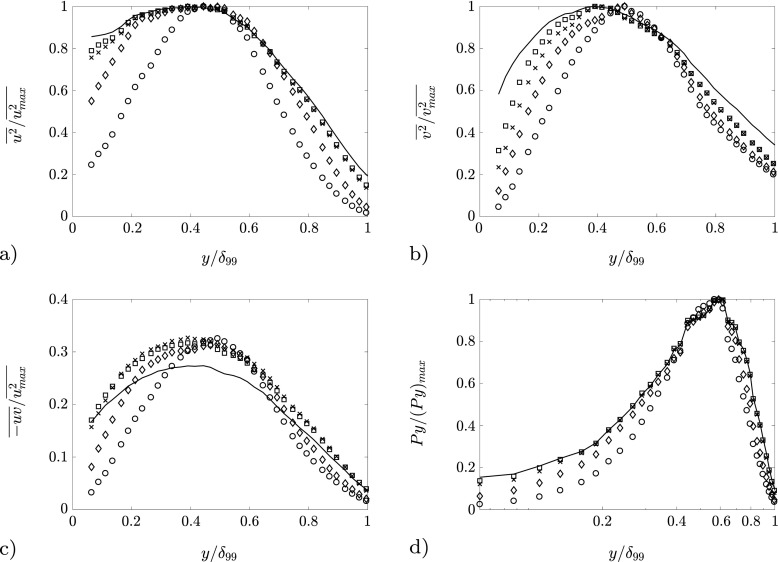
Comparative plot of profiles reconstructed with POD modes for *β* = 1.3at *R* *e*_*τ*_ = 1010. **a** Streamwise Reynolds normal stress; **b** Wall-normal Reynolds normal stress; **c** Reynolds shear-stress; d) Pre-multiplied turbulence production. The number of modes used in the reconstruction is represented with the following legend: (∘) 1 mode, (◇) 2 modes, (×) 4 modes, ($\square $) 50 modes and solid line 1150 modes

**Fig. 13:**
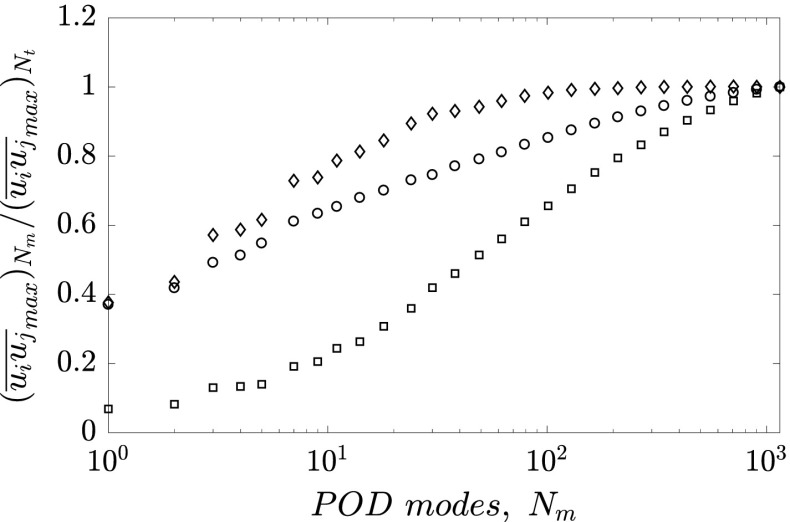
Comparative plot of the maximum value of the Reynolds stresses reconstructed with POD modes for *β* = 1.3at *R* *e*_*τ*_ = 1010. (∘) Corresponds to streamwise normal Reynolds stress, ($\square $) to wall-normal Reynolds stress and diamond symbols (◇) to Reynolds shear stress. Values are normalized with the maximum Reynolds stress value

Figure 12 shows the profiles of $\overline {u^{2}}$, $\overline {v^{2}}$, *P* *y*(which is the pre-multiplied turbulence production) and $-\overline {uv}$. The first three quantities are normalized with their respective maximum value obtained from the reconstructed profile. The Reynolds shear stress $-\overline {uv}$ is instead normalized with the maximum $\overline {u^{2}}$ from the reconstructed profile; this choice is due to the fact that the two-point correlation matrix $\underline {\underline {C}}$ does not take into account the covariance of *u* and *v*. The profiles are reported for *N*_*m*_ = 1, 2, 50, 1150 (the latter corresponds to the ensemble of all modes and represents the complete statistics). Consistently with the observations from modes 1–4, the first mode is already able to locate the fluctuation peaks, thus showing that the wall-normal locations of the maxima of Reynolds stresses and turbulence production are highly influenced by the large-scale features. The peak position is slightly adjusted by the pair formed by the second and the fourth mode, while all the following modes contribute practically uniformly to the generation of Reynolds stresses and turbulence production. The latter statement is further confirmed by the observation of Fig. 13, which shows that the maxima constantly increase with increasing number of modes. The peaks in the streamwise and Reynolds shear-stress profiles are mostly due to large-scale motions, whereas the peak in the wall-normal Reynolds stress is mostly due to smaller-scale features. In the case of the streamwise Reynolds normal stress it is also clear that the highest-order modes contribute in building up the inner peak of the variance profile. The first two POD modes contribute in building up the Reynolds-stress distributions more strongly at *y*/*δ*_99_ ≈ 0.4, which is within the outer layer. Adding more modes spreads out the distribution, but the peak location is unaffected. Interestingly, the production profiles peak at approximately the same location, i.e. *y*/*δ*_99_ ≈ 0.4, when using only the first mode, while the inclusion of more modes (and thus of small-scale contributions) shifts the peak toward larger wall-normal positions. This might be indicative of an interaction between small-scale and large-scale structures in producing turbulence in APG TBLs.

## Conclusions

In this work a study on APG TBLs under different Reynolds-number and pressure-gradient conditions has been carried out using PIV measurements which were supplemented by APG TBL LES data. The combination of both datasets allowed us to cover a wide Reynolds-number range: 2300 $<Re_{\delta ^{*}}<$ 34000.

Different PIV approaches for the measurement of turbulence statistics have been assessed against hot-wire measurements. Ensemble PTV with polynomial fits, as proposed in Ref. [49], has shown superior performances, with an excellent agreement also in second-order turbulence statistics from the wake region down to *y* ^+^ ≃ 10 for the TBL at the highest Reynolds number tested. In-plane Reynolds stresses have thus been estimated with ensemble PTV, allowing to assess experimentally the effect of the APG on the various components of the Reynolds-stress tensor. The increase of *β* is accompained by the strengthening of the wake and by a larger velocity defect, together with the appearance of an outer peak in the streamwise Reynolds stress profile at 500 ≤ *y* ^+^ ≤ 700and of a peak, approximately at the same location, of the Reynolds wall-normal and shear stresses. The experiments at matched *β* with different values of the Reynolds number show that the main effect is to displace these peaks farther away from the wall (when scaled in wall units) without altering significantly the peaks intensities while, conversely, changing *β* at fixed *R* *e*_*τ*_has little effect on the peak location and strong effect on the peak magnitude. This is also evident from inspection of the turbulence production profile.

LES data with matched *β* but different flow history support the conclusion that the turbulence statistics are significantly affected by the streamwise evolution of *β*. For instance, for a decreasing *β*, the boundary layer exhibits features of an effectively larger *β* (stronger peak intensity), while for increasing *β* the opposite occurs. This suggests that APG TBL features should be interpreted in terms of the accumulated effect of *β*(for instance, defining an average of the streamwise *β* evolution as in Ref. [57]), rather than in terms of the local value of *β*. This result is further supported by the analysis of turbulence production, which increases for larger values of *β*, while the production peak is moved toward higher *y* ^+^ for increasing *R* *e*_*τ*_. Equation 2, commonly used to estimate the turbulence production, has been assessed both with LES and PIV data, showing that the terms related to streamwise flow derivatives are non-negligible for cases at lower Reynolds number and greater *β*. Also in this case the accumulated *β* history represents an important parameter, more than the local value.

POD is used to show the effect of the large scales on the flow features. The energy eigenspectrum of the POD modes is apparently not affected by the Reynolds number nor by *β* in the ranges under study. The most energetic modes reflect the interaction between the outer and near-wall regions. In particular, the first POD mode represents a sweep or an ejection depending on the sign of the time coefficient in a certain snapshot. Q4 events are connected to high-speed-flow coherent motions and Q2 events to low-speed-flow ones, both being mirror images of one another. The mode organization is however affected by both the pressure gradient and the Reynolds number, and in agreement with Ref. [23] sweeps/ejections are moved farther from the wall. The contribution of the modes to the Reynolds stresses and turbulence production is analyzed by reconstructing these quantities with different numbers of modes. Our results show that the first mode is able to reconstruct the outer peak and reproduce the location of the fluctuation peak, while the following modes slightly adjust the position of the peaks and contribute to build the inner peak.
